# The renoprotective effect of d-limonene against cisplatin-induced acute kidney injury: targeting Nrf2/HO-1 and NLRP-3 inflammasome signaling pathways

**DOI:** 10.1007/s11010-026-05518-w

**Published:** 2026-04-28

**Authors:** Nourelhuda A. Mohammed, Amira Osman, Ehab S. Taher, Mohie Mahmoud Ibrahim, Bahaa Mohammed Badr, Salma M. Eraky, Fares E. M. Ali, Eman AbdelRaouf Mohamed

**Affiliations:** 1 Department of Physiology, Faculty of Medicine, Zagazig University, Zagazig, 44511 Egypt; 2 Department of Anatomy and Histology, Faculty of Medicine, Mutah University, Karak, 61710 Jordan; 3 Department of Histology and Cell Biology, Faculty of Medicine, Kafr Elsheikh University, Kafr Elsheikh, 33516 Egypt; 4 Department of Basic and Clinical Medical Sciences, Faculty of Dentistry, Zarqa University, Zarqa, 13110 Jordan; 5 Department of Pharmaceutical Organic Chemistry, Faculty of Pharmacy, Al-Azhar University, Assiut Branch, Assiut, 71524 Egypt; 6 Department of Basic Health Sciences, College of Applied Medical Sciences, Qassim University, Buraydah, 52555 Saudi Arabia; 7 Department of Anatomy and Embryology, Faculty of Medicine, Mansoura University, Mansoura, 35516 Egypt; 8 Department of Medical Microbiology and Immunology, Faculty of Medicine, Al- Azhar University, Assiut, 71524 Egypt; 9 Department of Biochemistry, Faculty of Pharmacy, Mansoura University, Mansoura, 35516 Egypt; 10 Department of Pharmacology and Toxicology, Faculty of Pharmacy, Al-Azhar University, Assiut Branch, Assiut, 71524 Egypt; 11 Michael Sayegh Faculty of Pharmacy, Aqaba University of Technology, Aqaba, 77110 Jordan

**Keywords:** Acute kidney injury, d-limonene, NLRP3 inflammasome, Nrf2/HO-1, Redox-sensitive pathways

## Abstract

Cisplatin is a potent chemotherapeutic agent that causes renal injury. d-limonene, a natural monoterpene, may confer renal protection. This study evaluated whether d-limonene pretreatment could mitigate cisplatin-induced nephrotoxicity. Wistar rats were divided into groups receiving vehicle, d-limonene (50 or 100 mg/kg), cisplatin (7.5 mg/kg, i.p.), or cisplatin plus d-limonene (50 or 100 mg/kg). d-limonene was administered orally once daily for 14 days, starting 7 days before and continuing 7 days after the single cisplatin injection on day 7, thus acting as both pre- and post-treatment (peri-cisplatin regimen). Cisplatin induced nephrotoxicity, shown by elevated serum urea, creatinine, uric acid, cystatin C, urine KIM-1, albumin/creatinine, and renal oxidative stress markers. It downregulated Nrf2/HO-1 and upregulated NADPH oxidase and NLRP3 inflammasome, with tubular damage histologically. d-limonene pretreatment dose-dependently improved renal dysfunction, oxidative stress, and suppressed NADPH oxidase, NLRP3, and IL-1β expression. High-dose d-limonene normalized most parameters, improved kidney histology, and renal somatic index. d-limonene mitigates cisplatin-induced nephrotoxicity with associated antioxidant and anti-inflammatory effects.

**Graphical abstract:**

The protective effect of d-limonene on cisplatin-induced nephrotoxicity through targeting oxidative stress (Nrf-2/HO-1 pathway) and inflammation (NLRP3 inflammasome)

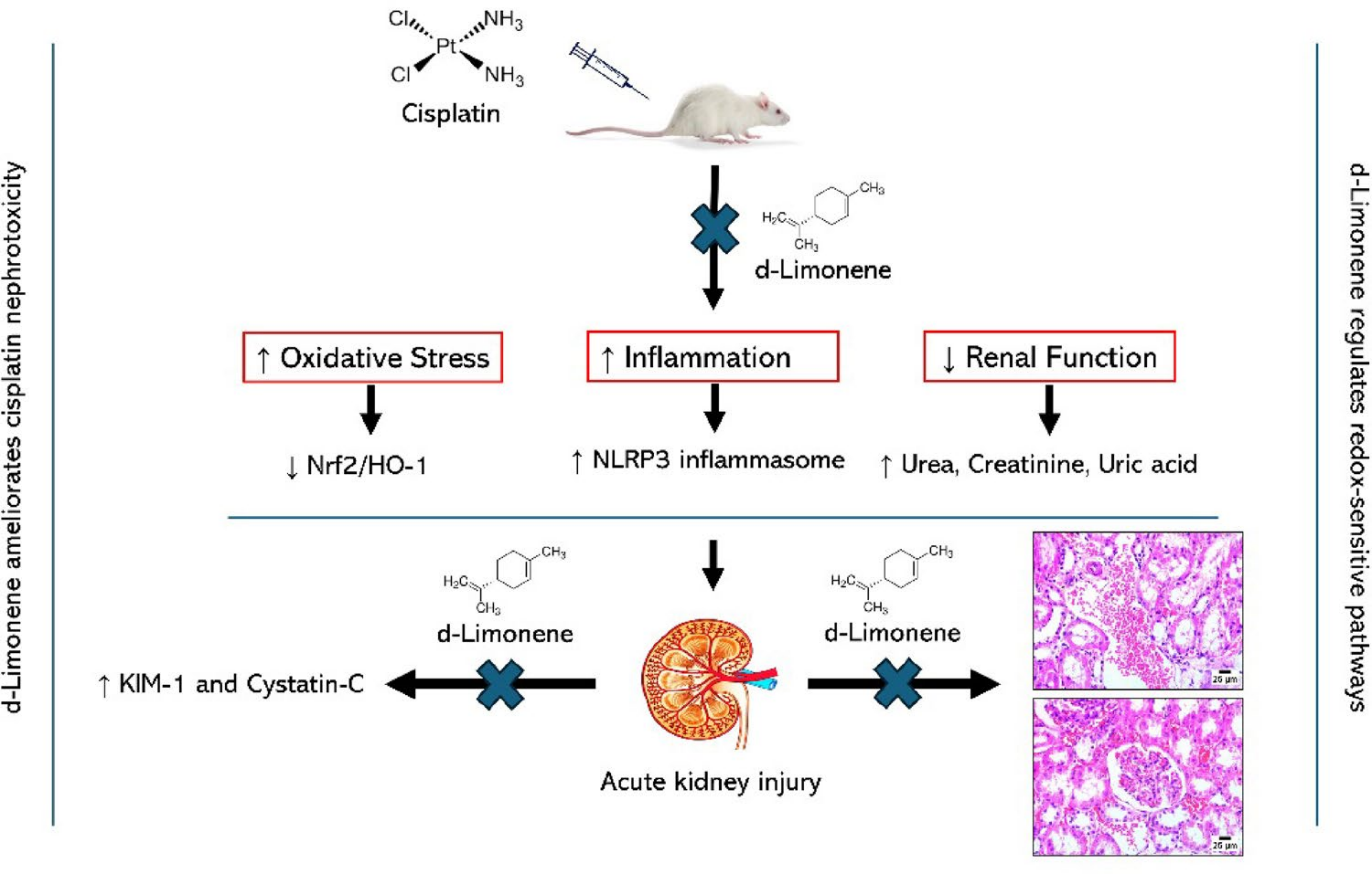

## Introduction

Acute kidney injury (AKI) is one of the most common adverse effects of chemotherapy with different medications. Chemotherapy medications cause up to 60% of all hospital-acquired AKI cases, and they are linked to significant morbidity and mortality [1]. Nephrotoxicity can arise through many processes, including direct cytotoxicity to the tubular epithelial cells, oxidative stress, DNA adducts, inflammation, and mitochondrial dysfunction [2].

Cisplatin is a widely used platinum-based chemotherapeutic agent effective against various solid tumors. However, its clinical utility is often limited by dose-dependent nephrotoxicity, which can lead to AKI ([3][4]. Cisplatin-induced AKI is characterized by reduced glomerular filtration and tubular damage, manifesting as elevated serum creatinine, blood urea nitrogen, and proteinuria [5]. Mechanistically, cisplatin accumulates in renal proximal tubular cells, triggering a complex pathogenic cascade involving oxidative stress, inflammation, and cell death [6, 7]. Besides, cisplatin promotes reactive oxygen species (ROS) directly through mitochondrial damage and upregulates enzymes like Nicotinamide Adenine Dinucleotide Phosphate (NADPH) oxidase while depleting endogenous antioxidants [8]. The resulting oxidative stress can damage lipids, proteins, and DNA, further aggravating renal injury. In parallel, cisplatin activates pro-inflammatory pathways.

Nuclear factor-κB (NF-κB) signaling is stimulated, leading to increased expression of cytokines such as tumor necrosis factor-α (TNF-α) and interleukins (IL) [9, 10]. Notably, the nucleotide-binding domain, leucine-rich–containing family, pyrin domain–containing-3 (NLRP3) inflammasome, a cytosolic protein complex that controls the maturation of IL-1β, has been implicated in cisplatin nephrotoxicity [11]. Studies have shown that cisplatin significantly elevates renal NLRP3, IL-1β, and IL-6 levels [12], contributing to an inflammatory response that exacerbates tubular cell injury. This inflammation-ROS cycle and subsequent apoptotic and necrotic cell death pathways collectively drive the AKI. Given that over a quarter of patients receiving cisplatin may develop some degree of kidney injury [6], there is a pressing need for renoprotective strategies.

The nuclear factor erythroid 2-related factor 2/hemoxygenase-1 (Nrf2/HO-1) pathway is a key defensive mechanism against oxidative renal injury. Nrf2 is a transcription factor that upregulates a battery of antioxidant and cytoprotective genes, including HO-1 and glutathione-synthesizing enzymes. Activation of Nrf2 has been shown to ameliorate cisplatin nephrotoxicity, whereas Nrf2 gene knockout exacerbates it [13]. Likewise, induced HO-1 can directly combat oxidative stress and inhibit pro-apoptotic signals in kidney cells, improving outcomes in AKI models [14].

d-limonene is a naturally occurring monocyclic monoterpene abundantly present in citrus peel oils (e.g., orange peel contains > 95% limonene among terpenoids) [15]. It is widely recognized for its broad bioactive properties, including antioxidant, anti-inflammatory, and antiapoptotic effects [16]. It has shown a protective impact in gentamicin-induced AKI in rats, improving renal function and histology while reducing oxidative stress and inflammation [17]. In addition, d-limonene has been reported to protect against several conditions, including cancer [18], neurotoxicity [19], cardiotoxicity [20], and diabetes [21].

In this study, we examined whether d-limonene mitigates cisplatin-induced AKI in rats, focusing on markers of Nrf2/HO-1 signaling and NLRP3 inflammasome activity.

## Materials and methods

### Drugs and chemicals

Cisplatin was purchased from MYLAN^®^ (50 mg/50 ml, ready for injection). d-limonene (purity > 97%) was purchased from Sigma-Aldrich (USA). All other chemicals were obtained from local certified sources of high analytical grade.

### Animals

Thirty adult male Wistar albino rats (12–16 weeks old) weighing approximately 200–250 g were obtained from the animal house at the Faculty of Medicine, Assiut University, Assiut, Egypt. The rats were housed under standard laboratory conditions (25 °C, 12:12 h light-dark cycle, *ad libitum* access to food and water). The institutional animal care committee at the Faculty of Pharmacy, Al-Azhar University, Assiut Branch, Assiut, Egypt, approved all treatments and dosing schedules, and experiments were conducted in accordance with ARRIVE 2.0 guidelines for animal research with approval number: AZ-AS/PH-REC/51/2024. The study was also approved by the Mansoura University Animal Care and Use Committee (MU-ACUC) with code number (PHARM.R.25.10.63). After acclimatization, rats were randomly allocated to the different experimental groups using a two-digit table of randomization. Investigators responsible for biochemical assays, qRT-PCR, Western blot densitometry, and histopathological evaluation were blinded to group allocation throughout data acquisition and analysis.

### Experimental design

After two weeks of acclimation, animals were randomly divided into five experimental groups (*n* = 6 per group). The Control group received the vehicles only (Saline for 14 days). The d-limonene control (100 mg/kg) group received d-limonene (100 mg/kg) alone without cisplatin [17]. d-limonene was administered once daily for 14 days, and on day 7, this group received a sham injection of saline instead of cisplatin. The cisplatin group received a single dose of cisplatin (7.5 mg/kg, intraperitoneal) on day 7 to induce AKI, and an oral vehicle was given daily for 14 days [22, 23]. The cisplatin + d-limonene (50 mg/kg) group received oral d-limonene at 50 mg/kg/day for 14 days, with a single intraperitoneal cisplatin injection (7.5 mg/kg) on day 7. d-limonene treatment continued for 7 days post-cisplatin. The cisplatin + d-limonene (100 mg/kg) group received oral d-limonene at 100 mg/kg/day for 14 days, with cisplatin (7.5 mg/kg, intraperitoneal) on day 7, and continuation of d-limonene through day 14. Accordingly, d-limonene was used as a peri-cisplatin regimen, encompassing both a 7-day pretreatment period before cisplatin administration and a 7-day early post-treatment period after cisplatin.

### Sample preparation

Twenty-four hours following the final treatment, rats’ weights were estimated, then rats were anesthetized using a combination of ketamine and xylazine administered intraperitoneally at doses of ketamine (75 mg/kg, i.p.) and xylazine (10 mg/kg, i.p.), and different samples were collected. Blood was drawn via cardiac puncture for serum preparation, and 24-hour urine was collected from metabolic cages on the final day. After collecting blood samples, sera were separated and used for renal function biomarkers. Kidney tissues were excised, rinsed in ice-cold saline, blotted dry, and weighed to compute the renal somatic index (RSI). Kidneys were sectioned into different parts to be used in various investigations. One kidney section was then homogenized in appropriate buffers for biochemical assays. Part of the kidneys were fixed in 10% neutral buffered formalin for at least 48 h and used for histopathological investigation. Another part was stored at −80 °C for qRT-PCR and western blot analyses.

###  Estimation of kidney function biomarkers

Serum urea (SPINREACT, CAT# Ref. 1001333), creatinine (SPINREACT, CAT# Ref. 1001110), and uric acid (SPINREACT, CAT# Ref. 1001013) levels were measured using colorimetric assay kits following the manufacturer’s instructions. Urine albumin (SPINREACT, CAT# Ref. 1001020) was measured using colorimetric assay kits following the manufacturer’s instructions. The urine albumin-to-creatinine ratio was calculated to assess proteinuria.

### Enzyme-linked immunosorbent assay (ELISA)

Urinary kidney injury molecule-1 (KIM-1; CAT# E-EL-R3019) and serum cystatin-C (CAT# E-EL-R0304) levels were quantified using a rat-specific ELISA kit (ELABSCIENCE, Wuhan, China), according to the manufacturer’s instructions. Briefly, urine or serum samples were diluted, added to the pre-coated ELISA plate wells in duplicate, and incubated at 37 °C for 90 min. After washing the plate 5 times with the supplied wash buffer, 100 µL of the provided biotinylated detection antibody was added to each well, followed by incubation at 37 °C for 60 min. After another washing step, 100 µL horseradish peroxidase-conjugated streptavidin solution was added, and the plate was incubated at 37 °C for an additional 30 min. Following thorough washing (5 times), substrate solution (100 µL) was added to each well and incubated in the dark at 37 °C for 15–20 min. The reaction was stopped by adding 50 µL stop solution per well. Absorbance was immediately measured at 450 nm using a microplate reader.

### Estimation of RSI

Kidney tissues were surgically isolated, washed in ice-cold saline, dried, and weighed to compute the RSI [24], according to the following equation:

$$RSI{\text{ }} = {\text{ }}[kidney{\text{ }}weight{\text{ }}(g)/Final{\text{ }}body{\text{ }}weight{\text{ }}(g)]{\text{ }} \times 100$$

### Histopathological examination

Fixed tissues from all groups were processed, embedded in paraffin, and sectioned at 4–5 μm thickness. Sections were stained with hematoxylin and eosin (H&E) for light microscopic examination of renal morphology [25]. A pathologist blinded to treatment groups evaluated the slides. Semi-quantitative scoring of renal damage was conducted [26]. Briefly, 10 non-overlapping kidney fields were examined per animal. Tubular epithelial degeneration/necrosis, tubular dilatation and intraluminal hyaline cast formation, interstitial hemorrhage and vascular congestion, and glomerular alterations were assessed. Each lesion was graded according to its extent within the field as follows: (–) none/normal (no detectable lesion); (+) mild changes involving < 25% of the field; (++) moderate involvement of 25–50% of the field; and (+++) severe involvement of > 50% of the field.

### Assessment of renal oxidative stress

We measured reduced glutathione (GSH) content in renal tissue homogenates using Ellman’s reagent method [27]. The inhibition of pyrogallol auto-oxidation assayed superoxide dismutase (SOD) activity in kidney homogenates [28]. Malondialdehyde (MDA), a marker of lipid peroxidation, was measured via the thiobarbituric acid reactive substances assay [29]. Renal hydrogen peroxide (H₂O₂) levels were determined using commercially available Biodiagnostic kit (Biodiagnostic Co., Cairo, Egypt), following the manufacturer’s instructions (CAT# HP 25).

### Gene expression (qRT-PCR) analysis

Total RNA was extracted from snap-frozen kidney tissue using TRIzol reagent according to the manufacturer’s instructions. cDNA was synthesized by reverse transcription of 1 µg RNA using a high-capacity cDNA kit. Quantitative real-time PCR was performed using gene-specific primers and SYBR Green on a PCR system. The mRNA levels of antioxidant and inflammatory pathway genes were measured, including Nrf2, HO-1, NLRP3, and IL-1β. GAPDH served as the housekeeping gene for normalization. The primers were listed in Table 1. Relative gene expression was calculated by the 2^^(−ΔΔCt)^ method [30], expressed as fold change relative to the control group. PCR conditions included an initial denaturation (95 °C, 5 min) followed by 35 amplification cycles (denaturation at 95 °C for 15 s, annealing/extension at 60 °C for 30 s).

**Table 1:** Primer sequences for the targeted genes

Target gene	Nucleotide sequence (5′−3′)
Nrf2	F: CTGGGCTGTGAATGAGGGAC R: TGTGAATGGCCGTGTGAAGT
HO-1	F: TGCTTGTTTCGCTCTATCTCC R: CTTTCAGAAGGGTCAGGTGTC
NLRP-3	F: GTGGCTACTCCCAGTGATTTGT R: TGCTTGCTTGGATGCTCCTT
IL-1β	F: GCACAGTTCCCCAACTGGTA R: GGAGACTGCCCATTCTCGAC
GAPDH	F: TGCTGGTGCTGAGTATGTCG R: TTGAGAGCAATGCCAGCC

### Western blot analysis

Portions of kidney tissue were lysed in RIPA buffer with protease and phosphatase inhibitors to extract total protein. The Bradford assay determined protein concentrations [31]. Equal amounts of protein (50 µg per lane) were separated by 10% SDS-PAGE and transferred onto PVDF membranes. The membranes were blocked with 5% non-fat milk and incubated overnight at 4 °C with primary antibodies against target proteins: Nrf2 (Catalog# YPA1865, dilution 1:1000), HO-1 (Catalog# YPA1919, dilution 1:500), NADPH oxidase (Catalog# E-AB-93356, dilution 1:1000), ASC (Catalog# YPA1695, dilution 1:500), and caspase-1 (Catalog# YPA2348, dilution 1:500). β-Actin (Catalog# E-AB-20031, dilution 1:3000) was used as a loading control. After washing, the membranes were incubated with appropriate ALP-conjugated secondary antibodies for 1 h at room temperature. Immunoreactive bands were visualized using a BCIP/NBT substrate (Catalog# BWR1067) and captured on an imaging system. Band densities were quantified by ImageJ software; target protein levels were normalized to β-actin and expressed relative to control. Uncropped, full-length images of the immunoblots are provided in Graphical abstract.

### Statistical analysis

Data were expressed as mean ± standard error of mean (SEM). Group comparisons were made by one-way ANOVA, followed by a post hoc Tukey’s test for multiple comparisons. A P-value < 0.05 was considered statistically significant. All analyses were performed using GraphPad Prism software (Version 10).

## Results

### Effect of d-limonene on cisplatin-induced AKI biomarkers

#### Effect on kidney dysfunctions

**Fig. 1:**
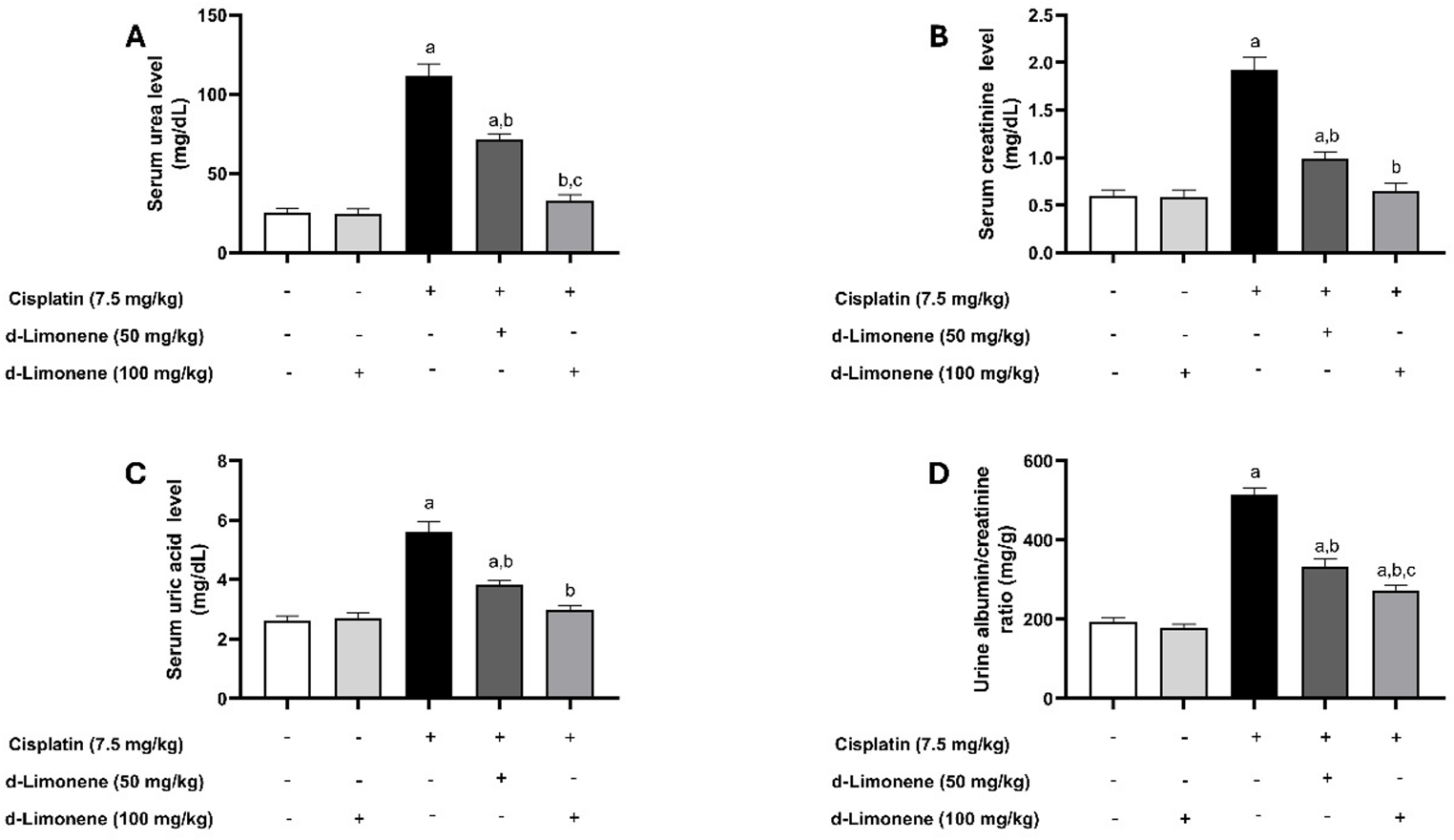
d-Limonene improves cisplatin-induced kidney dysfunction. **A** Serum urea, **B** serum creatinine, **C** serum uric acid, and **D** urine albumin/creatinine ratio in control (vehicle), d-Limonene-alone (100 mg/kg), cisplatin-alone, cisplatin + d-Limonene (50 mg/kg), and cisplatin + d-Limonene (100 mg/kg) groups. Bars represent mean ± SEM (*n* = 6). **a** = significantly different from control; **b** = significantly different from cisplatin; **c** = significantly different from cisplatin + d-Limonene (50 mg)

As expected, cisplatin administration led to a significant deterioration of renal function compared to the control group. Serum urea, creatinine, and uric acid levels were significantly elevated in cisplatin-treated rats compared to the normal control rats (Fig. 1A-C). These changes confirm the induction of AKI, as accumulating nitrogenous wastes reflect impaired glomerular filtration. Cisplatin also caused substantial proteinuria, evidenced by a high urine albumin/creatinine ratio (Fig. 1D). This indicates a significant protein loss in urine due to tubular damage or glomerular leakage. Crucially, d-limonene significantly mitigated these deleterious changes in a dose-dependent manner. Rats receiving cisplatin + d-limonene at 50 mg/kg showed lower serum urea, creatinine, and uric acid than cisplatin-alone. The 50 mg/kg dose also reduced the urine albumin/creatinine ratio (Fig. 1D). The high dose d-limonene (100 mg/kg) afforded greater protection, such as serum urea, creatinine, uric acid, and urine albumin/creatinine ratio compared to the cisplatin control group (Fig. 1). These results show that d-limonene mitigated declines in renal excretory function and reduced proteinuria in cisplatin-treated rats, with the 100 mg/kg dose being more effective.

#### Effect on KIM-1 and cystatin-C levels

**Fig. 2:**
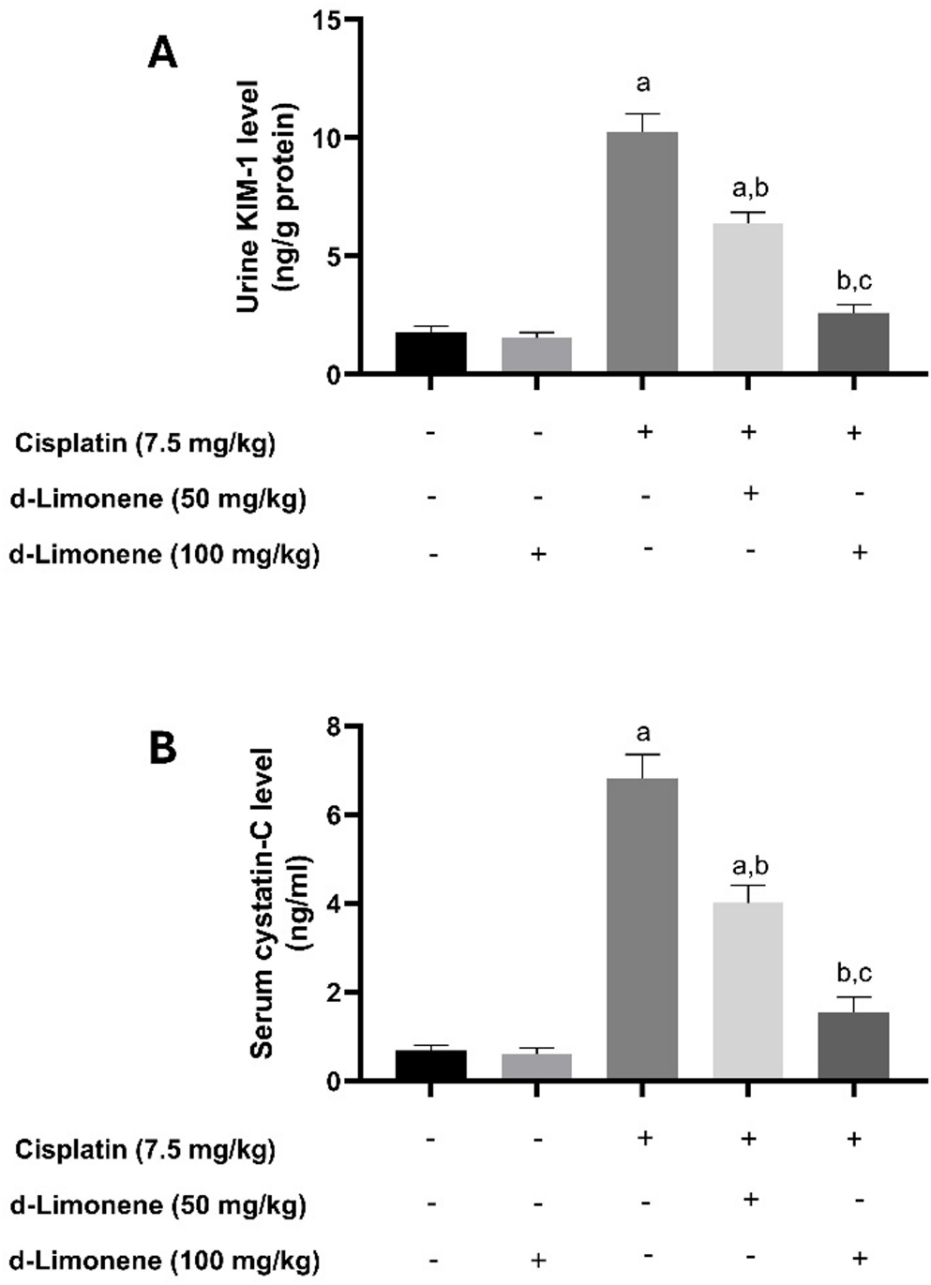
d-Limonene reduces cisplatin-elevated kidney injury biomarkers. **A** Urinary KIM-1 levels (ng/g urinary protein) and **B** Serum cystatin C (ng/mL) in the experimental groups. Bars represent mean ± SEM (*n* = 6). **a** = significantly different from control; **b** = different from cisplatin; **c** = different from cisplatin + d-Limonene (50 mg)

To further evaluate kidney injury, we measured KIM-1 and cystatin C levels. Cisplatin alone caused a marked increase in urinary KIM-1 and serum cystatin C compared to the normal control (Fig. 2), findings consistent with tubular injury and reduced glomerular filtration. d-limonene co-treatment significantly blunted these increases. Both d-limonene doses (50 and 100 mg/kg) significantly lower KIM-1 and cystatin C levels than the cisplatin control group (Fig. 2). These results align with the creatinine and urea findings and suggest that d-limonene attenuated cisplatin-induced kidney injury at functional and structural levels.

### Effect of d-limonene on renal somatic index (RSI) change after cisplatin challenge

**Fig. 3:**
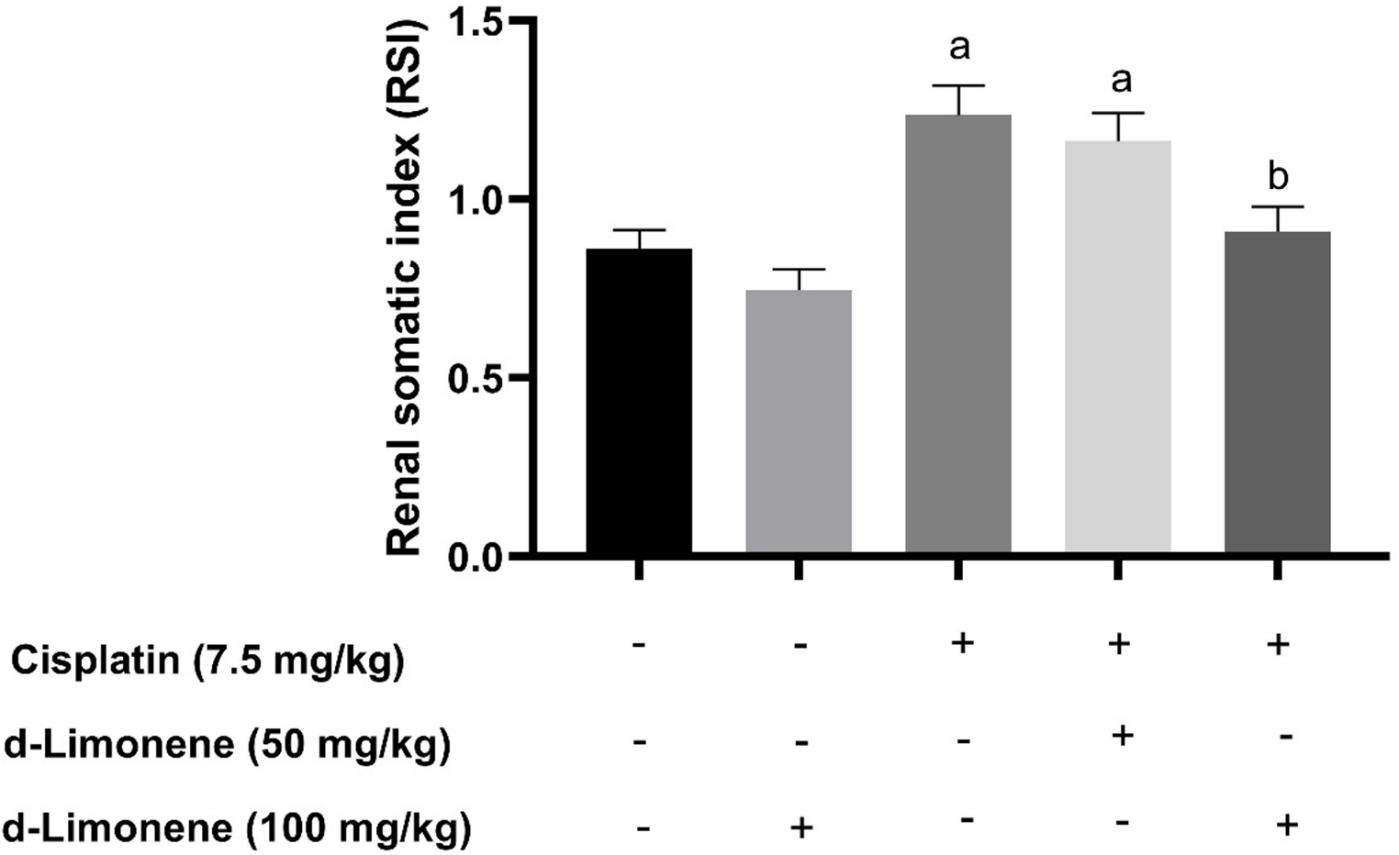
Effect of d-Limonene on renal somatic index (RSI) in cisplatin-induced AKI. Values represent the mean ± SEM (*n* = 6). **a** significantly different from control, and **b** significantly different from cisplatin alone

The kidney weight-to-body weight ratio provides an index of organ swelling or atrophy. In cisplatin-alone rats, RSI was significantly increased compared to the normal control, indicating renal enlargement likely driven by renal inflammation, edema, and cellular hypertrophy in response to injury. d-limonene treatment in high dose (100 mg/kg) significantly lowers RSI than cisplatin alone (Fig. 3). These findings suggest that d-limonene attenuates cisplatin-related pathological kidney enlargement.

### Effect of d-limonene on histopathological changes of cisplatin-induced AKI

**Fig. 4:**
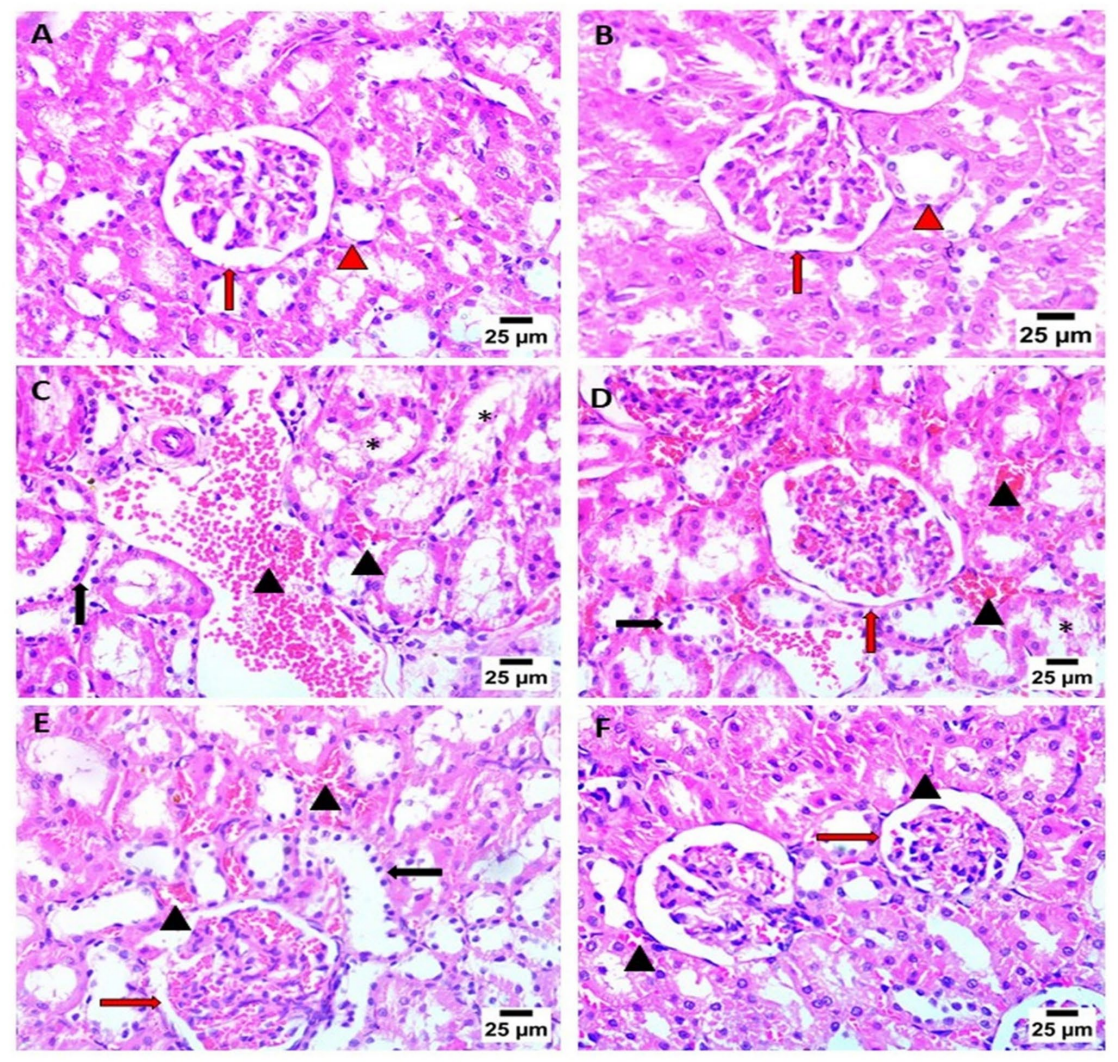
Photomicrographs of H&E-stained kidney Sect. (400× magnification, scale bar 25 μm). **A** The control group showed normal renal architecture with intact glomeruli and tubules. **B** d-Limonene 100 mg/kg alone, also showing normal histology. **C** and **D** The Cisplatin group displays severe tubular injury (black arrow: tubular necrosis and cast formation; red arrow: glomerular contraction; asterisks: inflammatory infiltrates). **E** Cisplatin + d-Limonene 50 mg/kg exhibits moderate damage (some necrotic tubules and casts remain, black arrowheads, but less than the cisplatin group. **F** Cisplatin + d-Limonene (100 mg/kg)-treated group demonstrating significantly improved renal histology with minimal pathological changes

The histopathological results of renal tissues in the present study (Fig. 4; Table 2) corroborated the biochemical findings. Control kidneys (Fig. 4A; Table 2) exhibited normal morphology, well-defined glomeruli, and renal tubules with intact epithelium. The kidneys of d-limonene alone (100 mg/kg) (Fig. 4B; Table 2) appeared similar to controls, indicating that d-limonene caused no structural damage to the kidneys. In contrast, cisplatin-alone kidneys (Fig. 4C; Table 2) showed severe pre-tubular blood vessels congestion, cytoplasmic vacuolations, and severe nuclear pyknosis in the lining epithelium of dilated renal tubules. Hypercellular glomerulus, periglomerular, and intraglomerular hemorrhage are indicated in this group. This pathology is characteristic of cisplatin nephrotoxicity, particularly affecting the S3 segment of proximal tubules in the outer medulla. Cisplatin + d-limonene (50 mg/kg) kidneys (Fig. 4D; Table 2) still showed tubular damage, but less severe than cisplatin alone; many tubules retained a more normal appearance. Glomeruli were largely normal in shape, though mild congestion persisted. Cisplatin + d-limonene (100 mg/kg) kidneys (Fig. 4E; Table 2) demonstrated substantial histological protection. Renal architecture was largely preserved; most tubules had intact epithelia, with only rare foci of cell damage, and glomeruli appeared essentially normal. Overall, the histopathological assessment clearly indicates that d-limonene, especially at 100 mg/kg, markedly attenuated cisplatin-induced structural kidney damage, correlating with the improvements seen in functional biomarkers.

**Table 2:** Semiquantitative analysis of pathological changes

Pathological lesions	Normal control	d-limonene control	Cisplatin control	Cisplatin + d-limonene 50	Cisplatin + d-limonene 100
Tubular damage	-	-	+++	++	+
Dilatation	-	-	++	++	-
Hemorrhage/congestion	-	-	+++	++	-
Glomerular changes	-	-	+++	+	-

(-); no change in the normal structure, (+) mild change, (++), moderate change, and (+++) severe change.

### Effect of d-limonene on kidney oxidative stress biomarkers after cisplatin challenge

#### Effect on cellular oxidant/antioxidant status

**Fig. 5:**
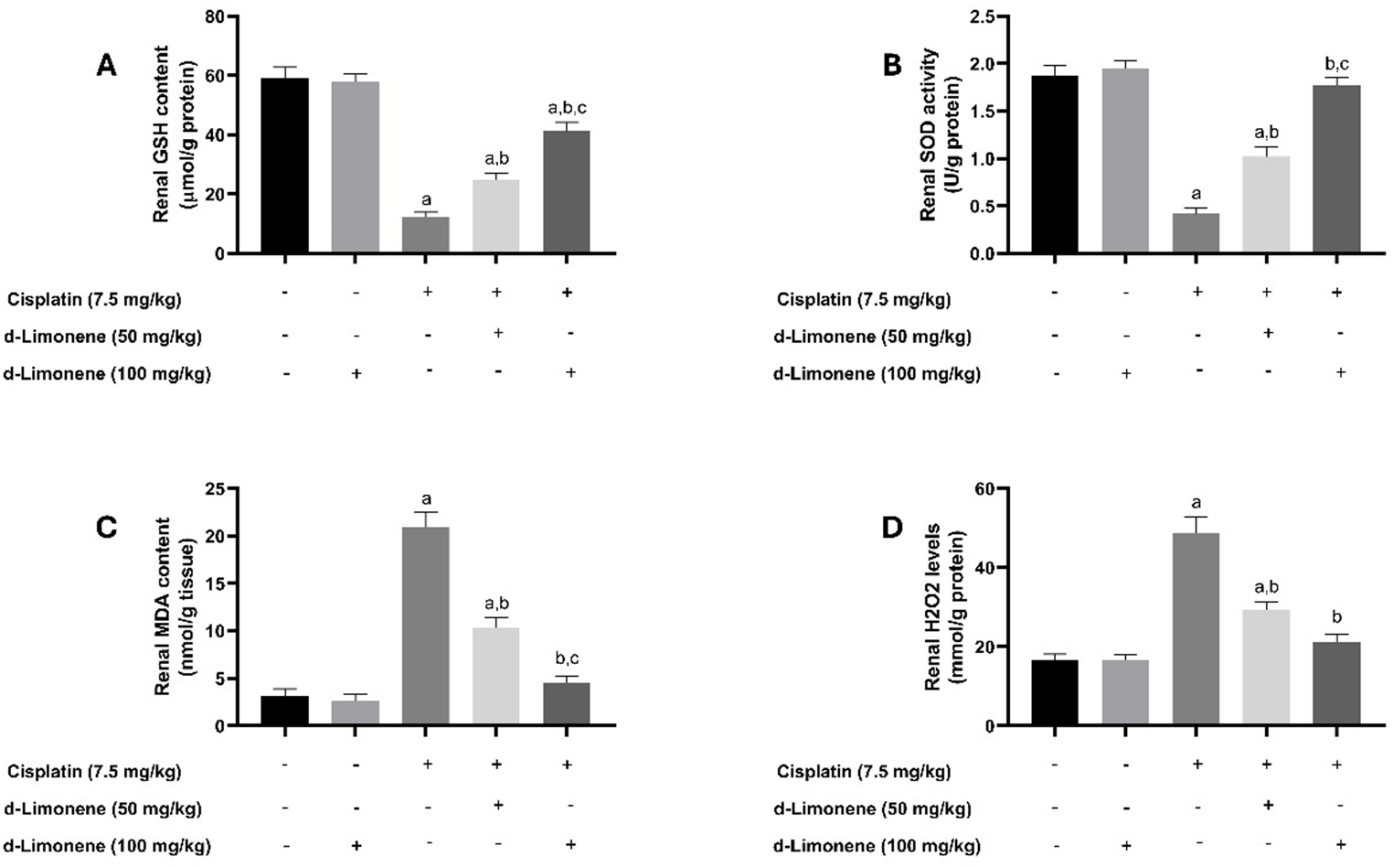
Effect of d-Limonene on renal oxidative stress markers in cisplatin-induced AKI. **A** Renal GSH content (µmol/g protein), **B** Renal SOD activity (U/µg protein), **C** Renal MDA content (nmol/g tissue), and **D** Renal H_2_O_2_ levels (nmol/g protein). Bars represent mean ± SEM (*n* = 6). **a** = significantly different from control; **b** = different from cisplatin; **c** = different from cisplatin + d-Limonene (50 mg)

Cisplatin-induced AKI was associated with severe oxidative stress in the kidney, as evidenced by significant reductions in GSH and SOD and concomitant increases in MDA and H_2_O_2_ compared to the normal control group (Fig. 5). On the contrary, d-limonene administration dose-dependently improved redox status, elevating GSH and SOD while lowering MDA and H_2_O_2_, indicating attenuation of oxidative injury (Fig. 5).

#### Effect on Nrf2, HO-1, and NADPH oxidase expression

**Fig. 6:**
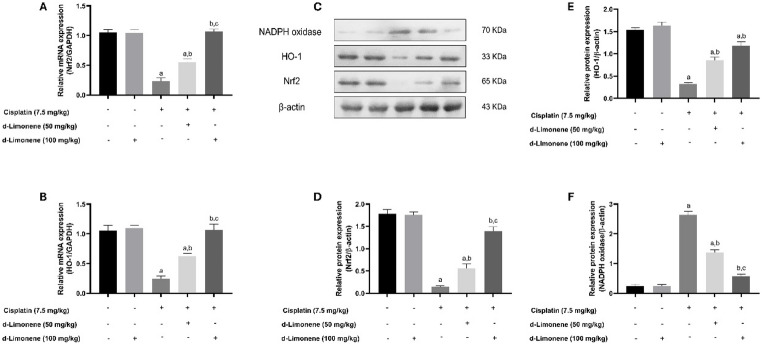
Effects of d-Limonene on the renal expression of oxidative stress-related markers in cisplatin-induced AKI. **A-B** Relative mRNA expression of Nrf2 and HO-1, respectively. **C** Representative western blot bands of Nrf2, HO-1, NADPH oxidase, and β-actin. **D-F** Quantitative protein expression of Nrf2, HO-1, and NADPH oxidase normalized to β-actin, respectively. Values represent mean ± SEM (*n* = 3–6). **a** significantly different from control, **b** significantly different from cisplatin alone, and **c** significantly different from d-Limonene (50 mg/kg) treatment

We examined Nrf2/HO-1 signaling and NADPH oxidase to clarify d-limonene’s mode of action. In cisplatin-injured kidneys, Nrf2 and HO-1 expression were markedly decreased compared to normal control rats, consistent with impaired antioxidant signaling, whereas NADPH oxidase was strongly upregulated compared to the normal control group (Fig. 6). d-limonene dose-dependently reversed these changes, elevating Nrf2 and HO-1 expression and suppressing NADPH oxidase expression compared to the cisplatin alone group (Fig. 6). These results support preservation of antioxidant defenses and attenuation of ROS production.

### Effect of d-limonene on kidney expression of NLRP3/IL-1β signal after cisplatin challenge

**Fig. 7:**
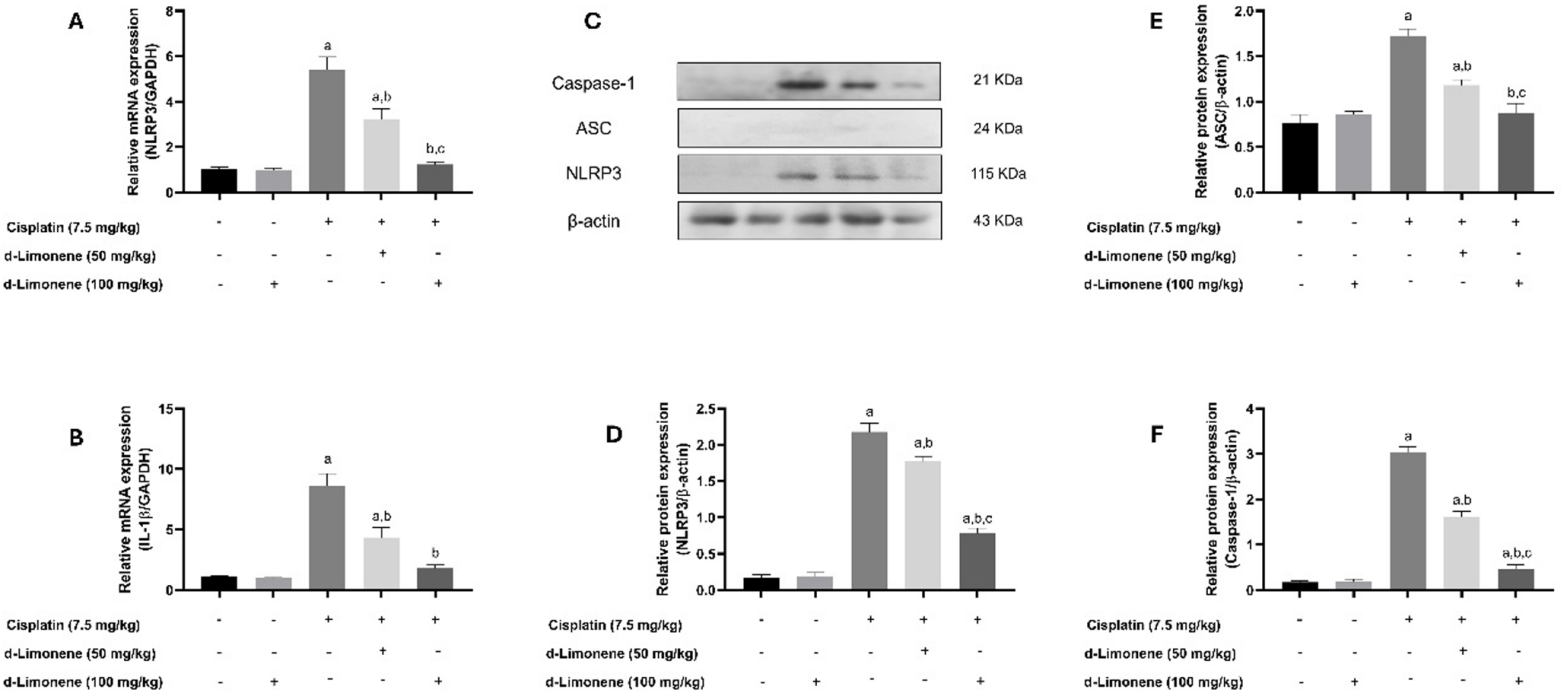
d-Limonene suppresses cisplatin-triggered inflammatory signaling and NLRP3 inflammasome activation in the kidney. **A** NLRP3 mRNA (fold change vs. control), **B** IL-1β mRNA, **C** Representative Western blots of NLRP3, ASC, caspase-1, and β-actin, **D** NLRP3 protein (relative to actin), **E** ASC protein, **F** Caspase-1 protein. Bars represent mean ± SEM (*n* = 3). **a** = significantly different from control; **b** = different from cisplatin; **c** = different from cisplatin + d-Limonene (50 mg)

As shown in Fig. 7A, NLRP3 mRNA levels in cisplatin-alone kidneys were significantly elevated compared to normal controls. Similarly, IL-1β mRNA, a downstream product of inflammasome activation, was significantly upregulated compared to normal control (Fig. 7B). These significant increases at the transcript level are consistent with activation of an innate immune/inflammatory response in the kidney. In parallel, western blot analysis revealed higher NLRP3 protein band intensity in cisplatin-treated rats compared to controls. The adaptor protein ASC was similarly increased in the cisplatin group compared to normal controls. Besides, the renal expression level of caspase-1 was strongly elevated by cisplatin compared to the control (Fig. 7F). In contrast, d-limonene in low and high doses significantly downregulated the NLRP3, ASC, caspase-1, and IL-1β expression in renal tissue compared to the cisplatin control group. These data demonstrate that d-limonene may mitigate cisplatin-induced NLRP3 inflammasome activation and the subsequent production of inflammatory mediator IL-1β in renal tissue.

##  Discussion

This study demonstrated that d-limonene exerts significant protective effects against cisplatin-induced AKI in rats. The data indicate that d-limonene, as a pretreatment and co-treatment, ameliorated renal dysfunction, blunted oxidative stress, and suppressed inflammatory signaling triggered by cisplatin.

Cisplatin nephrotoxicity is classically reflected by increases in serum creatinine and urea, as the drug causes acute tubular necrosis, leading to reduced glomerular filtration rate [3]. In our cisplatin-only group, creatinine and urea rose substantially, which aligns with clinical and experimental observations [3, 32]. d-limonene co-treatment significantly prevented these increases. Particularly at 100 mg/kg, d-limonene almost completely normalized creatinine and urea levels, suggesting near-preservation of glomerular filtration rate. Additionally, cisplatin caused marked proteinuria (albuminuria) and elevated cystatin C and KIM-1 levels, sensitive markers of kidney injury. KIM-1 is known to be upregulated in damaged proximal tubular cells and released into urine early in AKI, and cystatin C rises in blood when filtration drops [5]. d-limonene dose-dependently effectively decreased serum creatinine, uric acid, urea, and urine albumin/creatinine ratio levels in cisplatin-induced AKI. The observed reduction in these markers with d-limonene treatment aligns with a study conducted by Abdel-Daim et al., [33], who reported alleviating renal dysfunctions induced by cisplatin upon administration of Citrus limonia oil. Similarly, d-limonene has been shown to mitigate gentamicin-induced AKI by enhancing antioxidant defenses and reducing oxidative stress markers [17]. High-dose d-limonene reduced urinary KIM-1 and serum cystatin C to near-control values, indicating substantial protection of tubular integrity and filtration capacity [33]. These improvements in functional and injury indices point to d-limonene mitigating the acute renal damage inflicted by cisplatin.

Under cisplatin treatment, the RSI showed a marked increase. According to Jana et al., [34], increased kidney weight after a cisplatin injection may be due to inflammation and edema [35]. Hyponatremia can also result from cisplatin-induced nephron damage because the proximal tubule reabsorbs 67% of all salt [2]. Cisplatin induces renal impairment because of its severe AKI [36]. d-limonene reduced RSI, supporting a potential anti-inflammatory effect.

The histological analysis of cisplatin-treated renal tissue showed severe pre-tubular blood vessel congestion. The lining epithelium of renal tubules exhibited cytoplasmic vacuolations and severe nuclear pyknosis with intraluminal casts in distal tubules. Hypercellular glomerulus, periglomerular, and intraglomerular hemorrhage were also noted. These results demonstrated the development of distinctive features of AKI, which are brought on by cisplatin and result in a form of death distinct from the typical necrotic apoptotic features [37]. The pathophysiology of cisplatin-induced AKI is complicated and has been connected to inflammation, vascular damage, oxidative and endoplasmic reticulum stress, cellular uptake and efflux, and apoptosis [38]. d-limonene dose-dependently effectively decreased renal tubules cytoplasmic vacuolations, nuclear pyknosis, and pre-tubular blood vessels congestion. The histological restoration observed with d-limonene treatment, including reduced tubular damage and inflammation, is consistent with findings where natural compounds like Panax ginseng extract attenuated cisplatin-induced renal tissue damage and improved histopathological outcomes [39].

Oxidative stress is a central mechanism in cisplatin-induced renal cell injury. Cisplatin generates ROS through direct enzymatic processes, e.g., via NADPH oxidase and mitochondrial dysfunction, and by depleting antioxidants [40, 41]. In the present study, we observed that cisplatin drastically lowered renal GSH content and SOD activity while elevating MDA and H₂O₂ levels, confirming oxidative injury to lipids and other macromolecules. d-limonene’s protective role was strongly evident in this context; it dose-dependently replenished GSH and SOD and curtailed MDA and H₂O₂ accumulation. This suggests that d-limonene either directly scavenges ROS and/or bolsters the cellular antioxidant systems. Indeed, d-limonene has been reported to increase activities of catalase, glutathione peroxidase, and SOD in toxin-challenged tissues [17]. Our findings are consistent with those reports and underscored d-limonene’s function as an antioxidant [42]. The restoration of GSH, a crucial intracellular free radical scavenger, by d-limonene is significant. GSH depletion by cisplatin is a critical event leading to oxidative damage and mitochondrial apoptosis in tubular cells. By maintaining higher GSH, d-limonene likely helped neutralize ROS and protect mitochondria [43].

A novel aspect of this study is demonstrating that d-limonene modulates the Nrf2/HO-1 pathway in cisplatin nephrotoxicity. Nrf2 is typically activated under oxidative stress, translocating to the nucleus to induce genes like HO-1 and GSH-related enzymes [44]. However, in severe AKI, Nrf2 responses may be insufficient or impaired [45]. Our results showed that cisplatin significantly downregulated Nrf2 and HO-1, which could exacerbate oxidative injury due to a blunted antioxidant response [3, 46]. Treatment with d-limonene prevented this suppression and upregulated Nrf2 and HO-1 levels, especially at 100 mg/kg. This suggests that d-limonene can activate Nrf2 signaling in the kidney. It is possible that d-limonene, through mild oxidative challenge or kinase signaling modulation, promotes Nrf2 stabilization and nuclear accumulation [17]. Enhanced HO-1 expression is beneficial as HO-1 can degrade pro-oxidant heme groups and produce biliverdin and bilirubin, which have antioxidant properties, as well as carbon monoxide, which has anti-apoptotic and anti-inflammatory signaling functions in stressed tissues [47]. Our findings with d-limonene align well with previous studies that indicate the importance of the Nrf2/HO-1 mechanism in AKI [3, 6, 46], suggesting a conserved pathway of renoprotection via Nrf2 activation. Moreover, the observed reduction in cisplatin-induced NADPH oxidase expression by d-limonene could be secondary to Nrf2 activation or direct interference with oxidase-inducing signals [6]. Lower NADPH oxidase means reduced superoxide generation, contributing to the lower H₂O₂ and MDA in d-limonene groups. Accordingly, Nrf2/HO-1 pathway activation seemed to have a critical role in d-limonene’s antioxidant effect and helped to maintain redox balance in renal cells under cisplatin stress.

Cisplatin injury is not only a consequence of direct ROS damage but is amplified by inflammation. Damaged tubular cells release danger signals that activate innate immunity pathways, including the NLRP3 inflammasome and NF-κB, leading to the secretion of cytokines and the recruitment of leukocytes [48]. IL-1β is a potent pro-inflammatory cytokine that can worsen tissue injury [49]. Our data show that cisplatin induced IL-1β and NLRP3/ASC/caspase-1 in kidney tissue, confirming inflammasome activation. d-limonene markedly blunted these changes, as evidenced by reduced IL-1β gene expression and lower protein levels of NLRP3, ASC, and active caspase-1. This indicates that d-limonene has a strong anti-inflammatory effect, likely by interfering with upstream signaling that triggers inflammasome assembly. One plausible mechanism is inhibiting NF-κB activation; the gentamicin-AKI study reported that d-limonene suppressed NF-κB and inflammatory cytokines [17]. Since NF-κB drives transcription of pro-IL-1β and NLRP3, its inhibition would result in lower inflammasome components [50]. Additionally, d-limonene’s reduction in ROS may directly impede NLRP3 activation, as oxidative stress is known to be a key trigger for the inflammasome. By maintaining redox homeostasis, d-limonene could create a cellular environment less permissive to NLRP3 activation by maintaining redox homeostasis. The net effect decreases IL-1β production and potentially less recruitment of neutrophils and macrophages to the kidney, as the histology shows reduced inflammatory infiltrates in d-limonene-treated rats.

Clinically, our results suggest that d-limonene may have potential as a nephroprotective adjunct to cisplatin chemotherapy, but this remains hypothetical at the current stage. Its oral availability and low toxicity are advantageous; however, translation to clinical practice will require rigorous evaluation in well-designed clinical studies.

This study has several limitations. First, although we observed changes in Nrf2, NLRP3, ASC, caspase-1, and IL-1β expression, we did not perform genetic or pharmacological manipulation of these pathways (e.g., Nrf2 inhibition/knockdown, direct NLRP3 activation, or use of pathway-specific blockers). Therefore, the proposed involvement of the Nrf2–NLRP3 axis remains correlative, and the present data should be viewed as hypothesis-generating rather than providing definitive mechanistic proof. Second, the work was conducted in a single experimental model of cisplatin-induced nephrotoxicity, which may limit the generalizability of the findings to other settings and to humans. Third, we used a relatively short follow-up period, so potential long-term effects on renal structure and function could not be fully characterized. Future studies incorporating targeted pathway modulation, additional nephrotoxicity models, and longer follow-up will be required to confirm and extend these observations.

## Conclusions

This study provides evidence that d-limonene has potent nephroprotective effects against cisplatin-induced AKI in a rat model. d-limonene significantly improved renal function and reduced tubular injury biomarkers. Mechanistically, d-limonene’s benefits are attributed to its antioxidant and anti-inflammatory actions; it mitigated oxidative stress by restoring GSH and SOD and by activating the Nrf2/HO-1 pathway, and it suppressed inflammatory responses by inhibiting the NLRP3 inflammasome and associated IL-1β release. These actions helped preserve renal cellular integrity, reflected in improved histopathology and reduced apoptosis/necrosis. Future clinical studies are warranted to evaluate efficacy and safety in humans (Graphical abstract). 

## Data Availability

No datasets were generated or analysed during the current study.

## Abbreviations

HO-1: Heme oxygenase-1
KIM-1: Kidney injury molecule-1
Nrf2: Nuclear factor erythroid 2–related factor 2
NLRP3: NOD-like receptor family pyrin domain containing 3
